# Video-based hand gesture recognition via SPD manifold spatial representation and optical flow motion features

**DOI:** 10.1371/journal.pone.0348122

**Published:** 2026-05-14

**Authors:** Zhonghai Bai, Václav Snášel, Seyedali Mirjalili, Bay Vo, Jeng-Shyang Pan, Lingping Kong, Crina Grosan

**Affiliations:** 1 Faculty of Electrical Engineering and Computer Science, VSB – Technical University of Ostrava, Ostrava, Czech Republic; 2 Center for Advanced Technologies and Engineering, CATEN, Ostrava, Czech Republic; 3 Centre for Artificial Intelligence Research and Optimisation, Torrens University Australia, Brisbane, Australia; 4 University Research and Innovation Center, Obuda University, Budapest, Hungary; 5 Faculty of Information Technology, HUTECH University, Ho Chi Minh City, Vietnam; 6 School of Artificial Intelligence, Nanjing University of Information Science and Technology, Nanjing, China; 7 Digital Health and Applied Technology Assessment, King’s College London, London, United Kingdom; 8 King’s Institute for Artificial Intelligence, King’s College London, London, United Kingdom; University of Marburg: Philipps-Universitat Marburg, GERMANY

## Abstract

Hand gesture recognition plays an important role in human–computer interaction, yet accurately modeling both spatial structure and temporal motion patterns in video-based vision systems remains challenging. Many existing approaches focus on either spatial appearance or motion information, which can limit their ability to capture the full complexity of dynamic hand gestures evolving over time. In this work, we present a unified feature representation framework that combines spatial descriptors modeled on the Symmetric Positive Definite (SPD) manifold with temporal motion features extracted from gesture video sequences using grid-based optical flow histograms in Euclidean space. Spatial covariance descriptors are mapped from the SPD manifold to a Euclidean space through the Log-Euclidean metric, enabling effective feature fusion while preserving intrinsic geometric properties. The resulting representation captures complementary spatial and temporal information in a compact and interpretable form. We evaluate the proposed framework on two publicly available video-based benchmark datasets for dynamic hand gesture recognition, the Cambridge Hand Gesture dataset and the Northwestern University Hand Gesture dataset. Experimental results demonstrate that the combined representation consistently improves classification performance compared to using spatial or temporal features alone, achieving 99.31% accuracy on the Cambridge dataset and 97.23% on the Northwestern dataset. These findings indicate that integrating manifold-aware spatial features with motion-based temporal cues provides a practical and effective solution for robust dynamic hand gesture recognition.

## Introduction

Hand gestures are a vital component of natural communication and a primary means of interaction for the hearing impaired. With advances in human-computer interaction and virtual reality technology, the field of gesture recognition has also developed rapidly. Involving movements of the fingers, hands, arms, head, face, or body, hand gestures offer an intuitive method to convey information. Despite significant progress, recognizing hand gestures remains challenging due to their complexity and variability. Robust hand gesture recognition systems are essential, given their wide range of applications. These include enhancing user experiences in virtual reality systems [1], contributing significantly to human-robot collaboration [2–4], playing a crucial role in sign language recognition [5,6], practicing music conducting [7], supporting learning and teaching assistance [8], etc.

Hand gesture recognition has attracted significant attention due to its wide range of applications and can be categorized into contact-based and vision-based systems. Contact-based approaches rely on physical devices (e.g., gloves or sensors), which may restrict movement and reduce user comfort. Vision-based systems, in contrast, allow more natural interaction and have become increasingly prominent, though achieving high accuracy remains challenging. In contemporary video-based recognition, methods generally rely on either traditional feature extraction or neural network–based feature learning. For example, support vector machine–based frameworks have been applied to general video classification tasks [9], while transformer-based architectures have shown strong potential for capturing complex visual interactions [10].

Following approaches used in general video-based recognition, video-based hand gesture recognition studies can be broadly categorized into traditional feature-based methods and neural network–based approaches. Traditional methods typically extract handcrafted spatial and temporal features from video frames; for example, Chen et al. [11] use real-time hand tracking with Fourier descriptors for spatial features and motion analysis for temporal dynamics, while Tang et al. [12] employ image entropy–based keyframe selection, followed by SURF and SIFT3D for spatial and temporal feature extraction. Neural network–based methods leverage deep architectures to jointly model spatial and temporal information, as in Feichtenhofer et al. [13], who integrate ConvNet streams spatially and temporally, and in Heidari et al. [14], who propose a 2DCNN-LSTM framework with improved keyframe extraction for dynamic gesture recognition.

However, many existing hand gesture recognition methods represent spatial features in Euclidean space, which may not explicitly preserve intrinsic geometric relationships when modeling covariance-based or region-level descriptors. In dynamic hand gesture classification, this limitation can affect the robustness of spatial representations, particularly in scenarios involving complex appearance variations across video frames. To address this issue, we adopt a Symmetric Positive Definite (SPD) manifold–based representation [15] to model spatial covariance features in a geometrically meaningful space. Using the Log-Euclidean metric, these manifold-based spatial descriptors are mapped into Euclidean space, enabling consistent integration with temporal motion features extracted via grid-based optical flow histograms. This representation facilitates coherent fusion of spatial and temporal information for dynamic hand gesture classification.

The main contributions of this paper are summarized as follows:

- **Complementary Spatial and Temporal Feature Modeling**: We present a unified framework that combines SPD manifold-based spatial covariance features with grid-based optical flow motion features, enabling the joint modeling of spatial structure and temporal dynamics in dynamic hand gesture recognition.
- **Log-Euclidean Mapping for Feature Fusion**: We employ the Log-Euclidean metric to map manifold-based spatial features into Euclidean space, allowing consistent integration with temporal motion descriptors within a unified representation.
- **Evaluation on Benchmark Datasets**: The proposed framework is evaluated on the Cambridge and Northwestern University hand gesture datasets, providing an empirical analysis of the effectiveness of combining spatial and temporal features for gesture classification.

The remainder of this paper is organized as follows. The Related work section reviews existing hand gesture recognition systems. The Methodology section presents our proposed framework, introducing the key components of our dynamic hand gesture recognition approach. Experiments and Analysis details the datasets, experimental setup, and performance evaluation. Finally, the Conclusion summarizes the main findings and outlines directions for future research.

## Related work

Hand gesture recognition systems can be classified into contact-based and vision-based categories, depending on whether they require physical devices. In our paper, we primarily focus on vision-based systems, which can be further categorized into two primary approaches: model-based and feature representation-based.

**Model-based methods** in gesture recognition involve constructing explicit mathematical or computational models that simulate the physical or geometrical properties of gestures. These methods typically start by minimizing a cost function that is derived from image cues such as edges [16], segmented silhouettes [17], or patch-based errors [18]. The cost function guides the adjustment of parameters within the hand model, such as joint angles and positions. The goal of this iterative process is to align the model’s projection with the observed image, thereby reducing discrepancies indicated by the cost function. This iterative refinement improves the model’s ability to accurately represent and recognize the observed hand gesture.

Recent advancements have further refined these model-based methods. Saremi et al. [19,20] enhance particle swarm optimization (PSO) in the development of techniques for accurately modeling hand postures, addressing the limitations of conventional gradient-based methods and enabling robust exploration of the solution space, thereby ensuring more reliable hand posture estimation in diverse scenarios. Complementing this approach, Boukhayma et al. [21] introduce an end-to-end deep learning method for predicting 3D hand shape and pose from RGB images in real-world settings. Their network combines a deep convolutional encoder with a fixed model-based decoder, utilizing an articulated mesh deformation model and a re-projection module for accurate 3D hand reconstruction.

**Feature representation-based methods** aim to extract spatio-temporal features from video frames, capturing both the visual characteristics and the motion dynamics of gestures from images or videos. Conventional approaches, such as that of Holte et al. [22], propose a view-invariant algorithm that identifies motion primitives in 3D data using 3D optical flow and harmonic motion context, applying a probabilistic Edit Distance classifier for gesture classification. Liu et al. [23] introduce a pioneering method that employs Genetic Programming(GP) to optimize 3D sequence-processing operators. This approach involves the random assembly of low-level 3D operators into tree-based combinations, which evolve iteratively through the GP system. Shen et al. [24] propose an approach leveraging motion divergence fields, Maximum Stable Extremal Regions (MSER)-based region detection, and local motion pattern descriptors. Their method includes efficient indexing with Term Frequency-Inverse Document Frequency (TF-IDF), resulting in high performance on large-scale gesture databases.

Deep learning approaches have also been utilized within the realm of feature representation-based methods for extracting spatio-temporal features. Sarma et al. [25] proposed a two-stream network with a 3D convolutional neural network (C3D) for gesture videos and a 2D CNN for optical flow motion templates (OFMT). C3D captures spatio-temporal information, while OFMT enhances recognition by providing additional motion details and filtering out irrelevant gestures, resulting in improved accuracy through stream fusion. Hou et al. [26] introduce the spatial-temporal attention residual temporal convolutional network (STA-Res-TCN) designed for skeleton-based dynamic hand gesture recognition. This model employs an attention mechanism to selectively emphasize informative spatial-temporal features while filtering out noise, thereby improving recognition accuracy. Miah et al. [27] propose a method that utilizes a multi-branch architecture comprising two graph-based neural network channels and a general deep learning channel. The two graph-based branches capture spatial-temporal features, while the third general deep learning branch extracts additional features. These features are then concatenated and processed through a fully connected layer for final classification. Liu et al. [28] propose a novel model that uses a two-branch shallow CNN to extract spatial features, which are then passed into a long short-term memory (LSTM) layer to capture the temporal features. Sahoo et al. [29] introduced the Densely Connected Residual Channel Attention Module (DRCAM) network, which features a cascading structure of residual blocks combined with a multiscale channel attention module to effectively capture both low- and high-level information related to hand gestures, while the cascading structures are interconnected through dense connectivity for enhanced feature propagation. Additionally, various other neural network-based approaches have been proposed for spatio-temporal feature extraction and gesture recognition [30–35], contributing to advancements in this field.

To provide a more comprehensive overview of recent advances in vision-based hand gesture recognition, we summarize representative methods from 2020 to 2025 in Table 1. The table includes both classical deep learning approaches and more recent transformer- and graph-based methods, highlighting their datasets, model architectures, and key advantages and limitations.

**Table 1:** Summary of representative vision-based hand gesture recognition methods (2020–2025).

Paper (Year)	Dataset	Model	Advantages	Limitations
Sarma et al. [25] (2020)	Palm’s Graffiti Digits, Custom dataset	two-stream CNN	spatio-temporal feature learning via motion fusion, improved robustness	high computational cost, complex architecture
Gao et al. [36] (2021)	Custom dataset	3D pose-based multimodal 3D CNN + ConvLSTM	effective RGB-D-skeleton fusion, improved dynamic gesture recognition accuracy	requires multi-modal inputs, high computational cost
Vysocky et al. [37] (2022)	Synthetic dataset	U-Net-based hand localization model	strong generalization without real data, suitable for industrial scenarios	sim-to-real gap, limited real-world robustness
Miah et al. [27] (2023)	MSRA, DHG, SHREC’17	multi-branch graph-based hybrid model with attention	rich skeleton representation via graph modeling and temporal fusion	complex architecture, high computational cost
Liu et al. [28] (2023)	Custom dataset	two-branch multimodal CNN-LSTM with deformable convolution	robust multimodal fusion, better generalization across views and subjects	requires multi-modal sensors, increased complexity
Sahoo et al. [29] (2023)	ASL-FS	CNN with densely connected residual attention (DRCAM)	multiscale feature learning with attention and feature reuse	increased model complexity
Zhou et al. [38] (2024)	OUHANDS, EgoHands	two-stage lightweight CNN (FGDSNet)	lightweight design with real-time performance	multi-stage training, moderate computational cost
Rekik et al. [39] (2024)	Custom dataset	LSTM-based temporal prediction model	captures temporal dependencies for real-time intention prediction	limited spatial modeling capability
Yu et al. [40] (2025)	PHOENIX-2014, PHOENIX-2014T, CSL, CSL-Daily	CNN-based CSLR with multi-scale temporal modeling	improved spatial–temporal representation via multi-scale modeling and attention	relies on CTC alignment, increased training complexity
Hubert et al. [41] (2025)	MuViH	YOLOv8 + ResNet multi-view framework	robust to occlusion via multi-view aggregation, improved accuracy	requires multi-camera setup, higher deployment cost

Most existing hand gesture recognition methods represent spatial features in Euclidean space, which may not explicitly preserve intrinsic geometric relationships, especially for covariance-based or region-level descriptors. This limitation can affect robustness in dynamic scenarios with complex variations across frames. To address this gap, we map SPD-based spatial descriptors via the Log-Euclidean metric into Euclidean space and integrate them with temporal features, enabling coherent spatial-temporal fusion for dynamic hand gesture classification.

## Methodology

Our methodology for hand gesture classification utilizes the Riemannian manifold properties of SPD matrices. Specifically, following the RieCovDs descriptor algorithm [42], we construct local feature vectors from each image, combining pixel coordinates, RGB values, and their spatial gradients. Using these features, we compute covariance matrices across image regions, forming SPD matrices that capture the intricate spatial characteristics inherent in hand gestures. By representing the hand gesture data on the SPD manifold, essential spatial relationships are preserved in a geometrically meaningful space. Furthermore, our approach innovatively combines these Riemannian manifold features with traditional features, enriching the feature representation with both spatial nuances and complementary aspects of the data. This fusion of geometrically informed SPD features with conventional features enables a holistic characterization of hand gestures, enhancing classification performance beyond traditional approaches.

**Fig 1:**
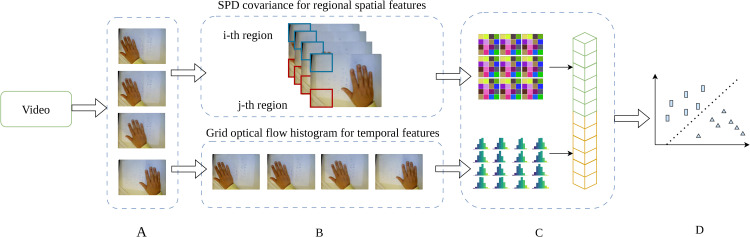
Overall architecture of the proposed framework. The pipeline consists of four main components: (A) keyframe extraction from input video; (B) feature extraction, including regional spatial features via SPD covariance matrices and temporal features via optical flow histograms on a grid; (C) feature unification and vectorization, where SPD features are mapped to Euclidean space and concatenated with temporal features; and (D) classification using the combined feature representation.

To provide a high-level overview of our proposed approach, we present the overall architecture in Fig 1, which illustrates the main components and their interactions. In the following sections, we provide detailed descriptions of each component of the methodology.

### SPD manifold construction

This section explores methods for generating SPD matrices in image analysis. It begins with the definition of the SPD manifold and then discusses conventional techniques for computing covariance matrices from image sets, focusing on pixel-wise correlations. It then introduces the Riemannian covariance descriptors (RieCovDs) [42] approach, which captures correlations between image regions using advanced Gaussian modeling and Riemannian geometry. This provides a more nuanced representation for improved feature extraction and classification.

#### SPD manifold overview.

A real-valued matrix *M* is classified as symmetric positive definite (SPD) if the quadratic form $v^{T}Mv>0$ for every non-zero vector $v\in {\mathbb{R}}^{d}$. The set of all *d* × *d* SPD matrices forms a commutative Lie group with a manifold structure denoted as $S_{d}^{++}$, defined in Eq (1):


$$\begin{array}{c} S_{d}^{++}=\left\{M\in {\mathbb{R}}^{d\times d}:M=M^{T},\;v^{T}Mv>0,\;\forall v\in {\mathbb{R}}^{d}⧵\{0\}\right\} \end{array}$$ (1)

This manifold is non-Euclidean, indicating a curved geometric structure. The SPD manifold is particularly significant in image analysis because it preserves intrinsic geometric relationships among data points, making it more suitable for tasks like feature extraction and classification compared to traditional Euclidean space.

#### Conventional covariance image set description.

In the context of image analysis, the SPD manifold offers a powerful representation for capturing the statistical characteristics and structural information present within image data.

A common approach involves representing image sets using covariance matrices, which are themselves SPD matrices. Each covariance matrix captures the pairwise relationships between pixel intensities across the image set, serving as a compact summary of the spatial correlations. By positioning these covariance matrices as points on the SPD manifold, we leverage its unique geometric structure to facilitate effective feature extraction and classification. To compute the covariance matrix for a set of images $I_{1},I_{2},\ldots ,I_{N}$, where *N* is the number of images, we first need to calculate the covariance between specified pixel positions across all images. Then, we use these covariance values to construct the covariance matrix.

To illustrate this calculation, let’s denote two positions within each image as *p* and *q*. The covariance of positions *p* and *q* can be expressed as:


$$\begin{array}{c} C_{p,q}=\frac{1}{N-1}\sum_{i=1}^{N}(I_{i,p}-\mu_{p})(I_{i,q}-\mu_{q}) \end{array}$$ (2)

where *I*_*i*,*p*_ and *I*_*i*,*q*_ denote the pixel intensities at positions *p* and *q* respectively in the *i*-th image, $\mu_{p}$ and $\mu_{q}$ are the means of pixel intensities at positions *p* and *q* respectively across all images. The covariance matrix representation can be written as Eq 3:


$$\begin{array}{c} C={\left[C_{p,q}\right]}_{p,q=1,2,\ldots ,d} \end{array}$$ (3)

where *C*_*p*,*q*_ computes by Eq 2 and *d* is the number of pixels in each image.

#### Riemannian covariance image set description.

In conventional image analysis, covariance matrices are typically computed to assess pixel-level correlations. However, an innovative approach, proposed in [42] and known as the Riemann covariance matrix descriptors (RieCovDs) method, diverges significantly from this convention. Instead of focusing solely on pixel-to-pixel correlations, RieCovDs captures correlations between distinct regions within images. This shift leads to a more discriminating representation of image sets, particularly advantageous for classification tasks. Below, we provide a detailed description of this method in Alg. 1.

**Algorithm 1 RieCovDs Descriptor Algorithm**

1: **Input**: A set of *n* images

2: **Output**: Riemannian Covariance Descriptors (Riemannian CovDs) characterizing the image collection

3: **Step 1: Region Partitioning:**

4:  Divide each image into *D* regions of uniform size.

5: **Step 2: Feature Extraction:**

6:  Extract pixel-wise features from the designated regions for each image.

7: **Step 3: Gaussian Modeling:**

8:  Model the feature vectors of each pair of regions (*i*-th and *j*-th) across the image collection using Gaussian distributions. This results in two sets of Gaussian models denoted as ${𝕏}_{i}=\chi_{i}^{1},\chi_{i}^{2},...,\chi_{i}^{n}$ and ${𝕏}_{j}=\chi_{j}^{1},\chi_{j}^{2},...,\chi_{j}^{n}$.

9: **Step 4: Covariance Computation:**

10:  Calculate the covariance $\text{cov}({𝕏}_{i},{𝕏}_{j})$ between the Gaussian models of ${𝕏}_{i}$ and ${𝕏}_{j}$ for each pair of regions (*i*-th and *j*-th).

11: **Step 5: Covariance Matrix Generation:**

12:  Generate the resulting covariance matrix *C* using the formula provided in Eq 11.

Now, let’s delve into the fundamental concepts underlying this algorithm.

**Gaussian Model:** The Gaussian model represents the distribution of local features within an image. Given a collection of *N* local features $F=\{f_{1},f_{2},\ldots ,f_{N}|f_{i}\in {\mathbb{R}}^{k}\}$, their distribution is modeled using maximum likelihood estimation with a Gaussian distribution, as shown in Eq 4:


$$\begin{array}{c} 𝒩(f_{i}|\mu ,\Sigma )=\frac{\exp\left(-\frac{1}{2}(f_{i}-\mu {)}^{T}\Sigma^{-1}(f_{i}-\mu )\right)}{\sqrt{\left(2\pi {)}^{k}\det(\Sigma \right)}}, \end{array}$$ (4)

where μ denotes the mean vector and Σ denotes the covariance matrix, calculated as $\mu =\frac{1}{N}\sum_{i=1}^{N}f_{i}$ and $\Sigma =\frac{1}{N-1}\sum_{i=1}^{N}(f_{i}-\mu )(f_{i}-\mu {)}^{T}$, respectively.

**Covariance of Gaussian Models**: To calculate the covariance between sets of Gaussian models, we first transform the problem into the task of computing Riemannian local difference vectors on the SPD manifold (RieLDV-S). This is achieved by embedding the Gaussian model into the SPD manifold, as expressed in Eq 5.


$$\begin{array}{c} 𝒩(\mu ,\Sigma )\to 𝒮=[\begin{array}{cc} \Sigma +\beta^{2}\mu \mu^{T} & \beta \mu \\ \beta \mu^{T} & 1 \end{array}], \end{array}$$ (5)

where β is a parameter that scales from 0 to 1. Faraki et al. [43] introduced a Riemannian local difference vector (RieLDV) formulation, which relies on geodesic distance and the gradient of geodesic distance functions, as represented in Eq 6.


$$\begin{array}{c} cov({𝕊}_{i},{𝕊}_{j})=\frac{1}{n-1}\sum_{p=1}^{n}\zeta (X_{p},E({𝕊}_{i}){)}^{T}\zeta (Y_{p},E({𝕊}_{j})), \end{array}$$ (6)

where $X_{p}$, $Y_{p}$ means SPD matrices achieved by applying Eq 5, ${𝕊}_{i}$ and ${𝕊}_{j}$ denote two sets of embedding matrices, $E({𝕊}_{i})$ denotes the expected value of ${𝕊}_{i}$, and the column vector $\zeta (\chi_{p},E({𝕊}_{i}))$ signifies the RieLDV relative to $E({𝕊}_{i})$.

The computation of ζ(𝒳p,E(𝕊)) relative to E(𝕊) is elucidated in Eq 7, where δ represents the geodesic distance on the curved manifold, and $\nabla_{E(𝕊)}\delta^{2}({𝒳}_{p},E(𝕊))$ stands for the gradient of the smooth distance function δ at point E(𝕊).


$$\begin{array}{c} \zeta ({𝒳}_{p},E(𝕊))=\delta ({𝒳}_{p},E(𝕊))\frac{\nabla_{E(𝕊)}\delta^{2}({𝒳}_{p},E(𝕊))}{\left\|\nabla_{E(𝕊)}\delta^{2}({𝒳}_{p},E(𝕊))\right\|} \end{array}$$ (7)

The Log-Euclidean Metric (LEM) on the SPD manifold is employed, and the gradients essential for computing RieLDV-S through Eq 7 are detailed in Eq 8 and Eq 9.


$$\begin{array}{c} \delta^{2}(X,Y)={\left\|log(Y)-log(X)\right\|}_{F}^{2} \end{array}$$ (8)

$$\begin{array}{c} \nabla_{X}\delta^{2}=X(log(X)-log(Y))+(log(X)-log(Y))X \end{array}$$ (9)

The expected value E(𝕏) of a set of SPD matrices is determined using the Fr’echet mean with LEM divergence, as expressed in Eq 10.


$$\begin{array}{c} E(𝕊)=exp(\frac{1}{m}\sum_{i=1}^{m}log({𝕊}_{i})) \end{array}$$ (10)

**Fig 2:**
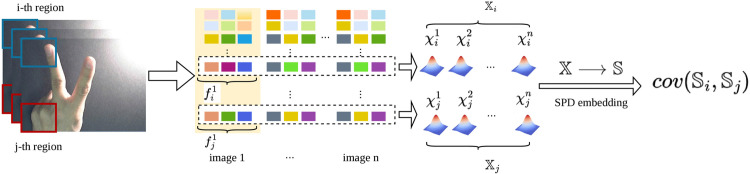
Region-based Covariance Matrix Computation for SPD Manifold Spatial Features.

Finally, the resulting covariance matrix ℂ is computed from individual covariances $\text{cov}({𝕊}_{i},{𝕊}_{j})$, as defined in Eq 11.


$$\begin{array}{c} \mathbb{C}=[{\mathbb{C}}_{i,j}{]}_{i,j=1,...,D}, \end{array}$$ (11)

where ${\mathbb{C}}_{i,j}=\text{cov}({𝕊}_{i},{𝕊}_{j})$. This methodological approach provides a robust characterization of image collections, contributing to various image analysis tasks. Fig 2 illustrates the process of computing region-based covariance.

### Motion detection

This section consists of two parts. First, we employ common methods to select keyframes from a video, and then we discuss the method we use to extract motion features from these keyframes.

#### Keyframes extraction strategy.

In this section, we will introduce three keyframe extraction methods: interval-based sampling, cluster-based sampling, and optical flow-based sampling.

**Interval-based sampling** is a simple but effective method for extracting keyframes in video analysis. This technique involves selecting frames at fixed intervals throughout the video to ensure an even distribution of keyframes. The keyframe extraction formula is given by Eq 12:


$$\begin{array}{c} KF_{idx}(i)=\frac{TF}{DKF}\times i,\;for\;i=0,1,\ldots ,DKF-1, \end{array}$$ (12)

where *KF*_*idx*_ denotes the index of the keyframe in the original video, “TF” represents the total number of video frames, and “DKF” denotes the desired number of keyframes. Interval-based sampling is computationally efficient because keyframes are selected at fixed intervals, but may not be effective in capturing important moments or major changes in content because it treats all frames equally. Interval-based sampling is well suited for tasks that require only basic summarization of video content and have limited computational resources.

**Cluster-based sampling** addresses the limitation of traditional methods, such as interval-based sampling, which may struggle to adequately summarize the variety of content in videos with a large number of frames. Cluster-based keyframe extraction consists of several steps. First, a feature vector ϕ(f) is extracted from each frame *f* in the video. Subsequently, these feature vectors are clustered into *K* clusters using algorithms such as K-means. Each cluster *C*_*k*_ contains frames with similar content features and its assignment is determined by Eq 13:


$$\begin{array}{c} C_{k}=\{f\in F\;|\;k=\arg\min_{j}\text{distance}(\phi (f),\mu_{j})\}, \end{array}$$ (13)

where $\mu_{j}$ denotes the centroid of the cluster *C*_*j*_, which is calculated as the average of the feature vectors within the cluster. The keyframes are selected according to Eq 14, where *f*_*k*_ denotes the frame closest to each centroid $\mu_{k}$:


$$\begin{array}{c} f_{k}=\arg\min_{f\in C_{k}}\text{distance}(\phi (f),\mu_{k}) \end{array}$$ (14)

Cluster-based keyframe extraction offers content-sensitive summarization by considering dynamic changes in video content, ensuring selected keyframes represent diverse segments. However, it may be computationally complex, requiring careful tuning of clustering algorithms and parameters for optimal results. Yet, it excels in tasks demanding detailed representations of video content, such as scene segmentation and content-based video summarization, effectively capturing diverse content dynamics while minimizing redundancy.

**Optical flow-based sampling** is also commonly employed to capture significant motion dynamics within video content. Optical flow vectors OF_*i*_ are computed between consecutive frame pairs $\left(f_{i},f_{i+1}\right)$, representing the apparent motion of objects. For each frame pair, the mean magnitude of optical flow MMOF_*i*_ is calculated as the average Euclidean norm of optical flow vectors, as given by Eq 15:


$$\begin{array}{c} {\text{MMOF}}_{i}=\frac{1}{H\times W}\sum_{x=1}^{W}\sum_{y=1}^{H}\|{\text{OF}}_{i}(x,y)\|, \end{array}$$ (15)

where *H* and *W* represent the height and width of the frames, respectively. Frames are ranked based on MMOF_*i*_, and the top K frames with the highest magnitudes are selected as keyframes. Specifically, the frame corresponding to the first frame in each pair is chosen as the keyframe.

While optical flow-based keyframe selection effectively identifies frames with notable motion transitions, it may overlook prolonged movements occurring gradually over several frames if the magnitude of motion is small in each pair. Additionally, sensitivity to abrupt changes in optical flow between consecutive frames can lead to inconsistent representation of motion sequences. Despite these limitations, this method is well-suited for tasks emphasizing dynamic temporal transitions, such as surveillance video analysis and traffic monitoring, where capturing significant motion dynamics is crucial.

#### Optical flow feature.

This section details the Gunnar-Farnebäck Optical Flow method for estimating pixel displacements using polynomial expansion and image pyramids. It also covers the estimation of grid-based histograms to capture motion information by partitioning the image, binning motion directions, and constructing histograms for further analysis.

**Optical Flow Estimation** utilizes the Gunnar-Farnebäck method [44], which applies polynomial expansion to approximate the intensity value *f*(*x*) of a pixel at position *x* within its neighborhood. This approach involves fitting a quadratic model to the pixel intensities, expressed as


$$\begin{array}{c} f(x)\sim x^{T}Ax+b^{T}x+c, \end{array}$$ (16)

where the coefficients *A*, *b*, and *c* are determined by the weighted least squares method to fit the grayscale values in the neighborhood. These coefficients capture the spatial relationships and intensity variations within the neighborhood. Assuming a theoretical case of pure translation, where no deformation or noise is present, the intensity value of a pixel at position *x* in the current frame *N* is identical to its intensity in the previous frame *N* − 1, after accounting for the displacement. This relationship is expressed as:


$$\begin{array}{c} f_{N}(x)=f_{N-1}(x-d), \end{array}$$ (17)

where *d* = (*d*_*x*_, *d*_*y*_) represents the translational displacement vector. By applying the quadratic polynomial model in Eq 16 to two consecutive frames and incorporating the translation assumption in Eq 17, the displacement vector can be derived as Eq 18:


$$\begin{array}{c} d=-\frac{1}{2}A_{N-1}^{-1}(b_{N}-b_{N-1}) \end{array}$$ (18)

The coefficients *A*_*N*−1_ and *A*_*N*_ are ideally expected to remain equal between two consecutive frames during ideal translation. However, in practice, the approximation


$$\begin{array}{c} A_{m}=\frac{1}{2}(A_{N-1}+A_{N}) \end{array}$$ (19)

is employed, as shown in Eq 19.

Although displacement information can be solved using the pointwise method, excessive noise often hinders the results. Therefore, the authors propose a more efficient approach: integrating information within pixel neighborhoods to solve for displacement under the assumption of gradual displacement changes. This method is described as follows:


$$\begin{array}{c} d=(\sum wA_{m}^{T}A_{m}{)}^{-1}\sum wA_{m}^{T}\Delta b_{m} \end{array}$$ (20)

$$\begin{array}{c} \Delta b_{m}=\frac{1}{2}(b_{N-1}-b_{N}) \end{array}$$ (21)

where *w* represents the weight function assigned to individual pixels within the neighborhood, typically a Gaussian or similar weighting function. The displacement *d* is therefore obtained as Eq 20, with $\Delta b_{m}$ defined in Eq 21.

The original algorithm designed for handling small movements faces challenges when confronted with large motions. To overcome this challenge, the Farneback optical flow algorithm integrates an image pyramid mechanism into its practical implementation. This addition enables the algorithm to effectively manage motion estimation across various scales with precision. This pyramid structure comprises multiple levels, each presenting a progressively lower resolution than its predecessor. Initially, tracking commences at the coarsest level, progressively advancing through the pyramid, meticulously refining point tracking across multiple resolutions. This strategic initiation at a lower resolution equips the algorithm to effectively handle larger point displacements between consecutive frames. As the tracking process unfolds across the pyramid levels, it systematically refines the estimated motion, thereby enhancing accuracy. Expanding the number of pyramid levels enables the algorithm to accommodate larger displacements between frames. However, it’s imperative to consider that this adjustment escalates the computational workload.

**Grid-based Histograms Estimation** involves estimating motion information through a three-step process:

Step 1: Grid Partitioning To capture spatial coherence and reduce the dimensionality of motion information, the image plane is partitioned into a grid of non-overlapping cells. Each cell serves as a spatial unit for aggregating motion information within its boundaries. The size of the grid cells can be adjusted based on the application requirements and computational constraints. Larger grid cells may provide a broader overview of motion patterns but might overlook finer details, whereas smaller grid cells offer higher spatial resolution but increase computational complexity.

Step 2: Binning In this step, the direction of motion θ is quantized into a predefined number of bins bin_num. Each bin covers an equal interval of 2π/bin_num. The quantization formula, as given by Eq 22, assigns each angle to its respective bin based on the angle’s value relative to the total range [0,2π):


$$\begin{array}{c} bin_{index}=\left\lfloor 2\pi *\theta /bin\_num\right\rfloor . \end{array}$$ (22)

This process discretizes the continuous range of motion directions into discrete segments, facilitating histogram construction to capture the distribution of motion orientations.

Step 3: Histogram Construction After binning, histograms are created for each grid cell. These histograms depict the frequency distribution of motion vectors within the cell. Each bin in the histogram corresponds to a specific range of motion values, with the height of each bin indicating the frequency of occurrence of motion vectors falling within that range.

**Fig 3:**
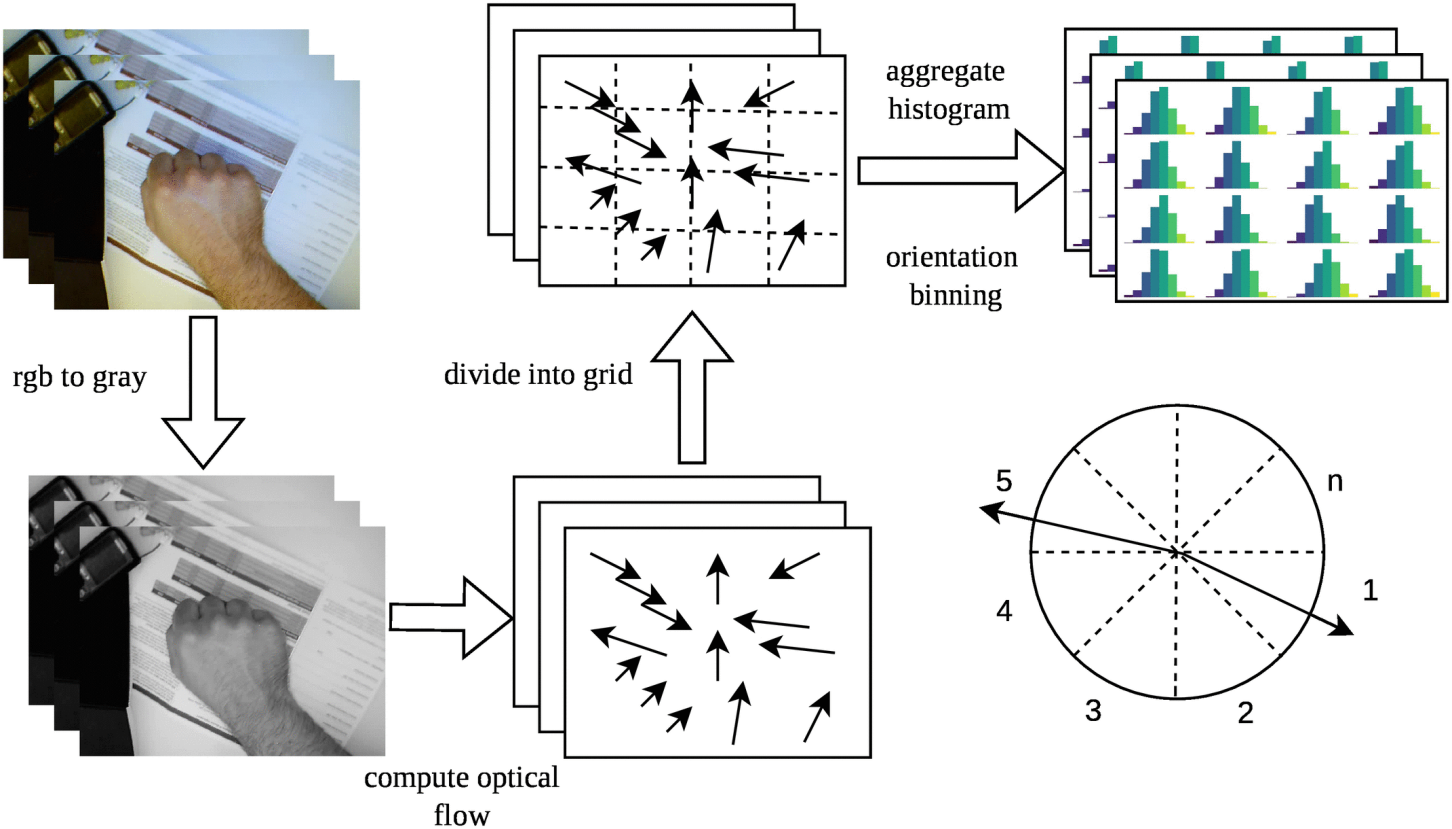
Grid-based Optical Flow Histogram Extraction for Temporal Features.

Fig 3 depicts the process, in which feature extraction of the spatial distribution of motion information in a video sequence, utilizing a grid-based histogram, aids in the further analysis and interpretation of potential dynamic changes.

### Feature fusion and classification

This section covers methods for mapping SPD matrices to Euclidean space, vectorizing features from tangent space and grid-based optical flow histograms, and combining these features for classification using an SVM.

#### Space mapping of SPD matrices.

The Riemannian manifold of SPD matrices has a complex, curved structure, making direct Euclidean computations inappropriate and often inaccurate. The tangent space at a point on this manifold provides a local Euclidean approximation, simplifying these computations.

For an SPD matrix *S*, the tangent space at *S* consists of all symmetric matrices that represent infinitesimal displacements from *S*. The Log-Euclidean Metric utilizes the matrix logarithm to map *S* to the tangent space at the identity matrix *I*, transforming the problem from the curved manifold to a flat Euclidean space.

The matrix logarithm operation is central to this mapping. For an SPD matrix *S*, the logarithmic map log:$S_{+}^{d}\to {𝒯}_{I}(S_{+}^{d})$ is defined by Eq 23:


$$\begin{array}{c} log(S)=Ulog(\Sigma )U^{T}, \end{array}$$ (23)

where $S=U\Sigma U^{T}$ is the eigenvalue decomposition of *S*, with *U* being the orthogonal matrix of eigenvectors and Σ the diagonal matrix of eigenvalues. The matrix logarithm log(Σ) is computed by taking the natural logarithm of each eigenvalue in Σ.

After the operation, the new matrix *log*(*S*) lies in the tangent space at the identity matrix *I*, which is in Euclidean space. Once mapped to the tangent space, SPD matrices can be processed using standard Euclidean methods and machine learning algorithms. This compatibility broadens the applicability and efficiency of existing techniques.

#### Feature vectorization.

For the tangent space feature, points in the tangent space can be represented minimally by considering the independent coefficients of symmetric matrices. According to Oncel et al. [45], a vector operator is defined at the identity matrix *I*, as given by Eq 24:


$$\begin{array}{c} vec_{I}(Y)=[y_{1,1},\sqrt{2}y_{1,2},\sqrt{2}y_{1,3},...,y_{2,2},\sqrt{2}y_{2,3},...,y_{d,d}{]}^{T}, \end{array}$$ (24)

where *y*_*i*,*j*_ represents the elements of the matrix *Y*.

For the optical flow feature, after computing the grid-based histograms of optical flow for each frame, we vectorize these histograms to create a feature vector suitable for further analysis or machine learning applications.

The vectorization process involves flattening the histograms of each grid cell into a single one-dimensional array and then concatenating these arrays to form a comprehensive feature vector for each frame. Subsequently, the feature vectors from all frames are concatenated to represent the entire video sequence. Optionally, these feature vectors can be standardized to ensure uniform contribution of each feature.

#### Feature concatenation and classification.

Once both the optical flow and tangent space features have been vectorized, they are concatenated to form a single comprehensive feature vector. This combined feature vector encapsulates both motion and structural information from the video.

The concatenated feature vectors are then used to train a Support Vector Machine (SVM) classifier. The SVM is chosen for its effectiveness in high-dimensional spaces and its ability to handle the combined feature set.

**Fig 4:**
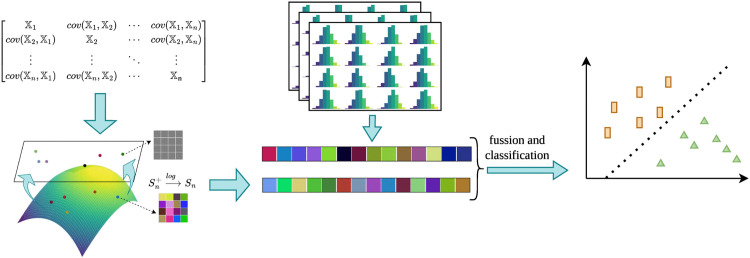
Illustration of the feature unification and classification process. Spatial features modeled on the SPD manifold are mapped to Euclidean space using Log-Euclidean metrics and then fused with temporal motion features extracted from optical flow histograms. The combined feature vector is used for robust hand gesture classification.

Fig 4 illustrates the process of feature matrix space mapping, feature vectorization, feature fusion, and classification. In this process, the spatial features extracted from the Riemannian manifold and the temporal features extracted from the Euclidean space are fused and used for classification.

## Experiments and analyze

This section presents the experimental evaluation and analysis of the proposed methods. We first introduce the datasets and experimental settings, and then provide parameter studies and performance evaluations to validate the effectiveness of the approach.

### Datasets and settings

**Fig 5:**
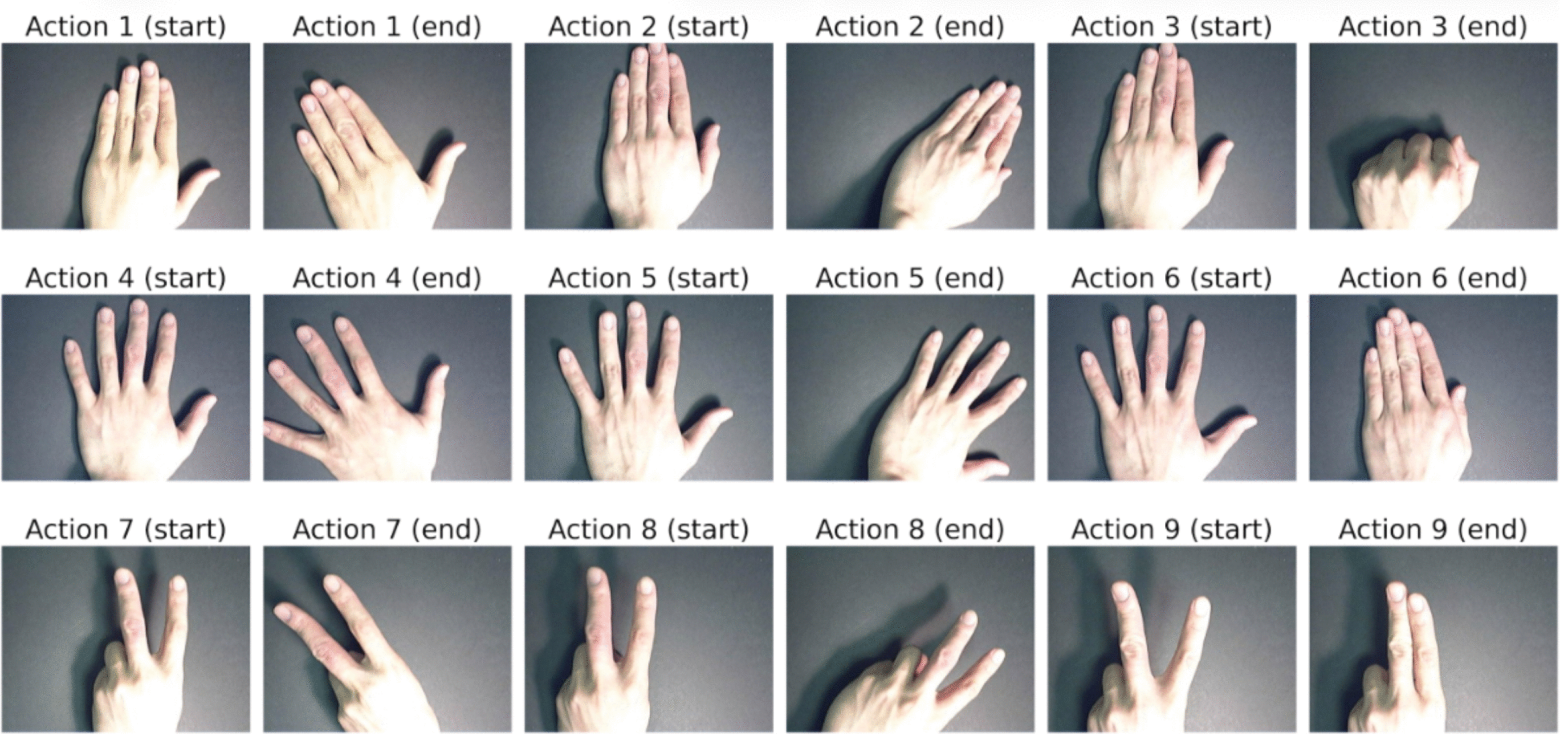
Cambridge hand gesture dataset [46]: combinations of three hand shapes (flat, spread, V-shape) with three motions (leftward, rightward, contract) for each shape.

**Fig 6:**
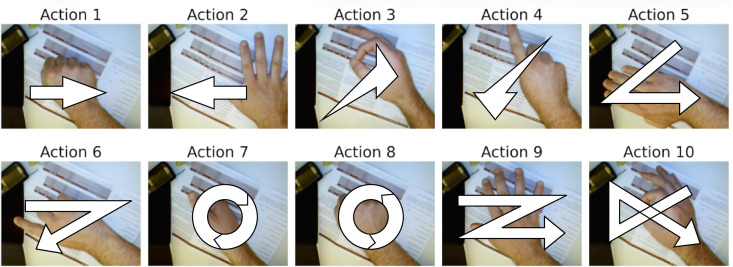
Northwestern University hand gesture dataset [24]: ten dynamic hand gestures including directional movements, rotations, circles, and symbolic gestures such as ‘Z’ and cross.

We evaluate the effectiveness of the proposed method on two publicly available benchmark datasets: the Cambridge Hand Gesture Dataset [46] and the Northwestern University Hand Gesture Dataset [24]. The motions represented in these datasets are illustrated in Fig 5 and Fig 6. Both datasets are publicly accessible online via https://github.com/Ha0Tang/HandGestureRecognition.

The Cambridge Hand Gesture Dataset [46] contains 900 image sequences at a resolution of 320×240 pixels. It includes 9 gesture classes, formed by combinations of 3 hand shapes (flat, spread, V-shape) and 3 motions (leftward, rightward, contract), as illustrated in Fig 5. Each class contains variations under 5 illumination conditions and 10 sequences per gesture, recorded from 2 subjects using a fixed camera setup. These sequences provide spatially and temporally isolated gestures suitable for evaluating dynamic hand gesture recognition methods.

The Northwestern University Hand Gesture Dataset [24] contains 1,050 short video sequences of 10 dynamic hand gestures, including directional movements, rotations, circles, and symbolic gestures such as ‘Z’ and cross, as illustrated in Fig 6. The videos were recorded at a resolution of 640×480 pixels from 15 subjects, with 7 sequences per gesture category. Each sequence includes variations across seven hand postures: Fist, Fingers extended, ‘OK’, Index, Side Hand, Side Index, and Thumb. This dataset provides a diverse set of spatial and temporal patterns suitable for evaluating dynamic hand gesture recognition methods.

For both datasets, we used a 60:40 train-test split, where 60% of the data was used for training and 40% for testing. This split ensures a balanced evaluation of the proposed method by providing sufficient samples for training while retaining enough data for testing.

### Parameter analysis

The algorithm proposed in this article is developed in Python. All experiments were conducted on macOS based on 4 Intel i7 2.2 GHz CPU cores and 16GB RAM.

In this subsection, we will first determine the keyframe selection method used in this study. Next, we will describe the parameters utilized in our method and provide justification for their selection. Finally, we will discuss the parameter tuning process employed to optimize performance and avoid overfitting.

#### Keyframe selection method.

In our framework, two primary parameters must be determined: the keyframe extraction method and the number of frames to extract from the gesture sequence, as described in the section “Motion detection.”

**Fig 7:**
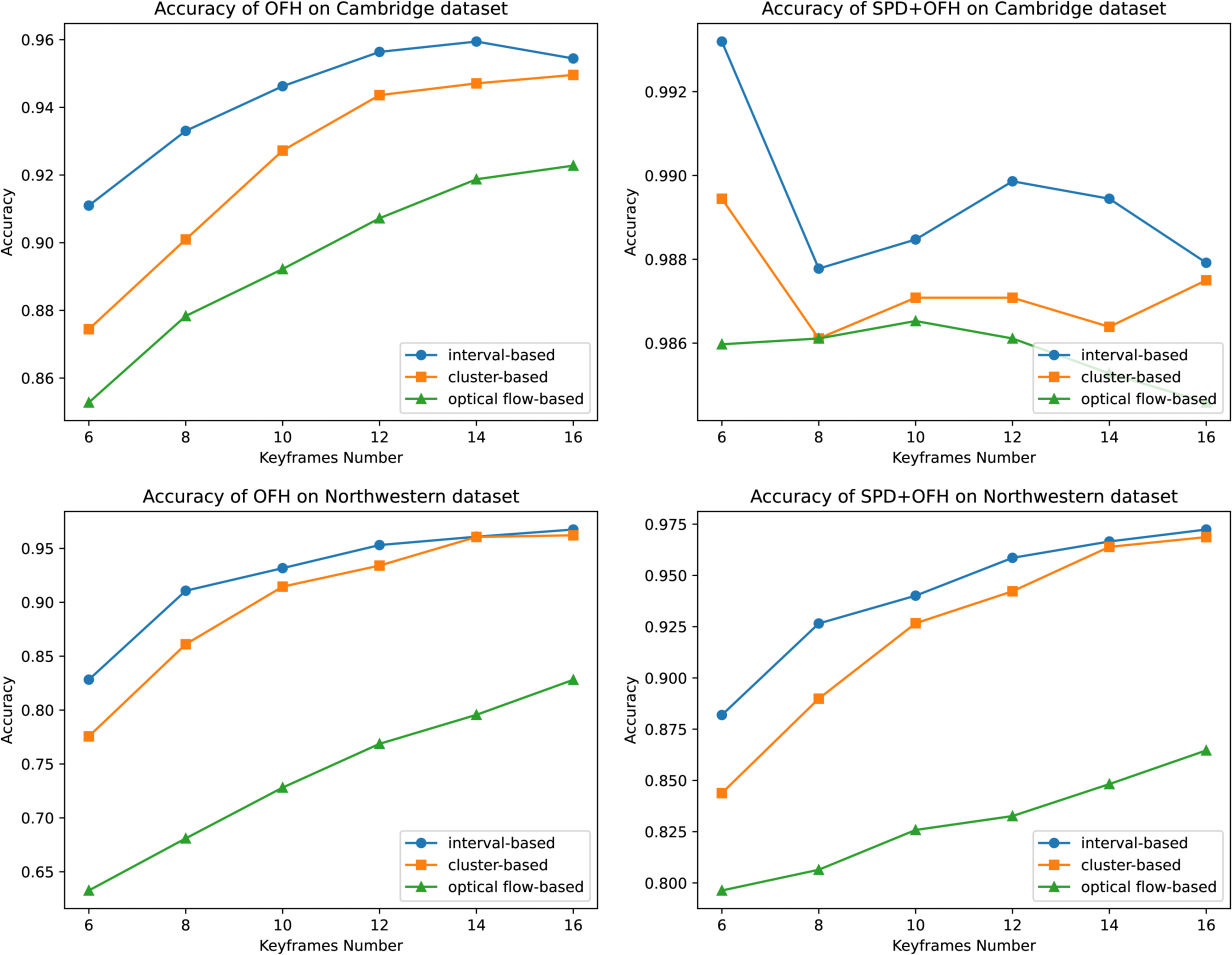
Classification accuracy for different keyframe selection methods.

Fig 7 shows the average performance of the test classification after running the experiment 20 times with different train-test split seeds. It is evident that the experimental performance of the “interval-based sampling method” for extracting keyframes outperforms the other two keyframe extraction methods on both datasets. This holds true whether the features extracted by the Grid-based Optical Flow Histogram (OFH) method are used alone for classification or in combination with the features extracted by the SPD matrices method.

The Fig 7 also reveals the effect of changing the number of keyframes on classification accuracy using different feature extraction methods across the Northwest and Cambridge datasets. In the Northwest dataset, there is a consistent and notable improvement in classification accuracy with an increasing number of frames, whether using OFH features alone or combined with SPD features. Conversely, the Cambridge dataset exhibits more nuanced patterns: while classification based on OFH features initially benefits from additional frames, this advantage levels off or slightly diminishes with the interval-based sampling method. Moreover, the fusion of OFH and SPD features achieves peak classification performance with 6 frames. Classification results fluctuated with an increasing number of frames but consistently remained lower than the accuracy achieved with the initial 6-frame configuration. This underscores that fewer frames are sufficient to distinguish actions in simpler datasets, such as Cambridge, where extracting too many features with additional frames may even lead to reduced accuracy, particularly in the fusion of OFH and SPD features. Notably, interval-based sampling consistently outperforms the other methods regarding classification accuracy, highlighting its effectiveness.

Besides accuracy, efficiency is also a crucial metric in evaluating these sampling methods. To provide a comprehensive assessment, we analyze their computational complexity. For cluster-based sampling, the primary computational task is K-Means clustering, which has a complexity of *O*(*T* × *N* × *K* × *D*), where T represents the number of iterations until convergence, N denotes the number of frames, K denotes the number of clusters, and D denotes the dimensionality of the feature space. Optical flow-based sampling involves calculating optical flow with a complexity of *O*(*N* × *H* × *W*), where N indicates the number of frames, and H and W denote the height and width of each frame, respectively. Interval-based sampling is the simplest, with a complexity of O(N), where N represents the number of frames. Theoretically, interval-based sampling is the most efficient. Empirically, for a hand gesture video with 61 frames, extracting 6 frames takes approximately 1.14 seconds for optical flow-based sampling, 0.12 seconds for cluster-based sampling, and 0.04 seconds for interval-based sampling. These results align with the theoretical complexities, indicating that interval-based sampling generally incurs the lowest computational cost, while optical flow-based and cluster-based methods are more resource-intensive, reflecting their respective complexities.

After analyzing and validating our experimental parameters, we selected the interval-based sampling method to extract keyframes, opting for 16 frames on the Northwest dataset and 6 frames on the Cambridge dataset.

#### Parameter description and justification.

To extract the local features required for RieCovDs, we generate an 11-dimensional local feature vector from the color image *I*, as given by Eq 25:


$$\begin{array}{c} \begin{array}{cc} F(x,y)=[ & x,y,I_{R}(x,y),\left|\frac{\partial I_{R}}{\partial x}\right|,\left|\frac{\partial I_{R}}{\partial y}\right|,I_{G}(x,y),\left|\frac{\partial I_{G}}{\partial x}\right|,\\ & \left|\frac{\partial I_{G}}{\partial y}\right|,I_{B}(x,y),\left|\frac{\partial I_{B}}{\partial x}\right|,\left|\frac{\partial I_{B}}{\partial y}\right|], \end{array} \end{array}$$ (25)

where *x* and *y* are the coordinates of a pixel. *I*_*R*_(*x*, *y*), *I*_*G*_(*x*, *y*), and *I*_*B*_(*x*, *y*) denote the color information at that pixel for the red, green, and blue channels, respectively. The gradients of the color information in the *x* and *y* directions are given by $\left|\frac{\partial I_{R}}{\partial x}\right|,\left|\frac{\partial I_{R}}{\partial y}\right|$ for the red channel, $\left|\frac{\partial I_{G}}{\partial x}\right|,\left|\frac{\partial I_{G}}{\partial y}\right|$ for the green channel, and $\left|\frac{\partial I_{B}}{\partial x}\right|,\left|\frac{\partial I_{B}}{\partial y}\right|$ for the blue channel. These gradients represent the first-order changes in the color information along the *x* and *y* axes. Additionally, the parameter β in Eq 5 is set as 0.7.

The parameters employed in the experiment are detailed in Table 2. For the OFH algorithm, we utilized the cv2.calcOpticalFlowFarneback implementation [44], which integrates a pyramid scheme for enhanced motion estimation. In the SPD algorithm, parameters such as *r*_*x*_, *r*_*y*_, *W*_*x*_, *W*_*y*_, *s*_*x*_, and *s*_*y*_ were employed to adjust the sliding window approach for extracting manifold features.

**Table 2:** Parameter settings for the algorithms used in this paper.

method	parameter explanation	value
OFH	parameter α means the scale employed to generate pyramids for each image	0.5
	parameter *L* means the number of pyramid layers	3
	parameter *W* means the averaging window size	15
	parameter *I* means the number of iterations at each pyramid level	3
	parameter *N* means the size of the pixel neighborhood employed to derive polynomial expansion for each pixel	5
	parameter σ means the standard deviation of the Gaussian applied for smoothing derivatives	1.2
	parameter *n*_bins_ means the number of bins used to quantize optical flow directions	8
	parameter *G*_*x*_ means the number of grid cells employed for partitioning the input frames along the horizontal dimension	8
	parameter *G*_*y*_ means the number of grid cells employed for partitioning the input frames along the vertical dimension	8
SPD	parameter *r*_*x*_ means scaling down the image along the horizontal dimension	240
	parameter *r*_*y*_ means scaling down the image along the vertical dimension	320
	parameter *W*_*x*_ means the size in pixels in the horizontal dimension of the sliding window	60
	parameter *W*_*y*_ means the size in pixels in the vertical dimension of the sliding window	80
	parameter *s*_*x*_ means the distance in pixels the sliding window moves horizontally at each step	30
	parameter *s*_*y*_ means the distance in pixels the sliding window moves vertically at each step	40

In the OFH method, the pyramid scale parameter (α = 0.5) is crucial for balancing the capture of fine motion details with computational efficiency. A lower scale (i.e., closer to 1) would increase computational costs with minimal improvements in accuracy, while a higher scale (i.e., closer to 0) could reduce the algorithm’s ability to capture small-scale motion details. The number of pyramid layers (*L* = 3) effectively captures motion at different scales. Three layers strike an optimal balance between capturing fine details and managing computational demands. The window size (*W* = 15) was selected to reduce noise while maintaining sensitivity to motion. Smaller windows resulted in noisier estimates, while larger windows caused over-smoothing, leading to a loss of crucial details. The grid sizes (*G*_*x*_ = 8, *G*_*y*_ = 8) were critical in determining the spatial resolution of the optical flow histograms. Properly chosen grid sizes ensured sufficient spatial detail without excessive computational overhead. Smaller grid sizes provided more detailed motion information but increased computational load and sensitivity to noise, while larger grid sizes reduced computational demands but might overlook finer motion details.

In the SPD method, the resizing dimensions (*r*_*x*_ = 240, *r*_*y*_ = 320) were selected to balance dimensionality reduction with the preservation of essential structural details, enhancing computational efficiency. The window sizes (*W*_*x*_ = 60, *W*_*y*_ = 80) were optimized to capture key features around each manifold point without incurring unnecessary computational costs. Smaller dimensions risked omitting relevant features, while larger ones could lead to redundancy. The step sizes for manifold sampling (*s*_*x*_ = 30, *s*_*y*_ = 40) were selected to ensure comprehensive image coverage while maintaining efficiency. Smaller steps might lead to redundant computations, whereas larger steps could miss significant features.

#### Parameter tuning and overfitting prevention.

To ensure the SVM classifier’s robustness and generalizability, we employed a combination of cross-validation and hyperparameter tuning strategies. Specifically, we used 5-fold cross-validation to evaluate the model’s performance and reduce bias. The training dataset was divided into five subsets, with each subset serving as the test set once, while the remaining subsets were used for training. This approach provided a reliable estimate of the model’s overall performance.

In conjunction with cross-validation, hyperparameter tuning was performed using grid search to optimize model complexity and mitigate overfitting. We systematically explored various values for the regularization parameter *C* (0.1, 1, 10, 100) and experimented with different kernel types (linear, polynomial, RBF, sigmoid). Special attention was given to the gamma parameter, which significantly influences the model’s decision boundary. To balance model complexity and performance, gamma was evaluated using two specific formulations that are dependent on the feature dataset.

Given the feature dataset $D=\left\{v_{1},v_{2},...,v_{n}\right\}$ obtained after feature extraction, where *n* denotes the number of records and each record vi is represented as a feature vector $v_{i}=\left[x_{i1},x_{i2},....,x_{im}\right]$, we evaluated gamma using two specific calculations, as given by Eq 26 and Eq 27:


$$\begin{array}{c} gamma_{1}=\frac{1}{\left|v\right|\cdot Var(D)} \end{array}$$ (26)

where *Var*(*D*) represents the variance of the feature dataset: $Var(D)=\frac{1}{\left|D\right|\cdot \left|v\right|}\sum_{i=1}^{\left|D\right|}\sum_{j=1}^{\left|v\right|}(x_{ij}-\mu )$ and μ is the mean of all *x*_*ij*_ values across the entire dataset.


$$\begin{array}{c} gamma_{2}=\frac{1}{\left|v\right|} \end{array}$$ (27)

By combining grid search with 5-fold cross-validation, we determined that *C* = 10, gamma set to Eq 26, and the RBF kernel provided an optimal balance, ensuring the model was well-tuned to avoid both overfitting and underfitting.

### Experiment evaluation

This section evaluates our classification model on the Cambridge and Northwestern datasets. We use four standard metrics: Accuracy, Precision, Recall, and F1 Score, defined in Eq 28–31. Our results demonstrate the competitive performance of the proposed approach compared to state-of-the-art methods.

#### Evaluates on four metrics.


$$\begin{array}{c} Accuracy=\frac{TP+TN}{TP+TN+FP+FN} \end{array}$$ (28)

$$\begin{array}{c} Precision=\frac{TP}{TP+FP} \end{array}$$ (29)

$$\begin{array}{c} Recall=\frac{TP}{TP+FN} \end{array}$$ (30)

$$\begin{array}{c} F1\;Score=2\times \frac{Precision\times Recall}{Precision+Recall} \end{array}$$ (31)

Here, TP, TN, FP, and FN denote True Positives, True Negatives, False Positives, and False Negatives, respectively.

#### Classification performance.

The precision, recall, and F1-score results, reported in Table 3, further confirm the effectiveness of the proposed method across both datasets. For this evaluation, the training and testing split was fixed using a random seed of 43 to ensure reproducible results.

**Table 3:** Precision, recall, and F1 scores for hand gesture classification on Northwestern and Cambridge datasets.

Dataset	Class	precision	recall	F1
Northwestern	move right	1.000	0.971	0.985
	move left	1.000	1.000	1.000
	rotate up	0.952	0.976	0.964
	rotate down	1.000	0.951	0.975
	move down-right	0.981	1.000	0.990
	move right-down	0.902	1.000	0.948
	clockwise circle	1.000	0.943	0.971
	counterclockwise circle	1.000	1.000	1.000
	“Z”	1.000	0.977	0.989
	“cross”	1.000	0.979	0.989
weighted avg		0.982	0.981	0.981
Cambridge	flat-leftward	1.000	1.000	1.000
	flat-rightward	0.975	1.000	0.987
	flat-contract	0.974	1.000	0.987
	spread-leftward	1.000	1.000	1.000
	spread-rightward	1.000	0.956	0.977
	spread-contract	1.000	1.000	1.000
	v-shape-leftward	1.000	1.000	1.000
	v-shape-rightward	1.000	1.000	1.000
	v-shape-contract	1.000	1.000	1.000
weighted avg		0.995	0.994	0.994

On the Northwestern dataset, most gesture classes achieve near-perfect performance. Minor deviations are observed in the “rotate down” class (recall of 0.951, F1-score of 0.975), as well as in “move right-down” and “clockwise circle,” reflecting slight variations in classification accuracy. The overall accuracy on the Northwestern dataset is 0.981, demonstrating that the model correctly classifies the vast majority of gestures.

Performance on the Cambridge dataset is even more consistent, with nearly all gesture classes attaining perfect scores. Only a slight reduction in recall is seen for “spread-rightward” (0.956). The overall accuracy on the Cambridge dataset is 0.994, indicating highly reliable classification across all gesture categories. The weighted average scores of 0.982 (Northwestern) and 0.994 (Cambridge) further underscore the robustness of the proposed method.

#### Ablation study.

To evaluate the contribution of each component in the proposed framework, we conduct an ablation study on SPD and OFH features, as shown in Table 4, comparing the performance of using SPD alone, OFH alone, and their combination. Each experiment was repeated 20 times, and the reported results correspond to the mean and standard deviation over these runs, providing a robust estimate of the model’s performance.

**Table 4:** Comparison of classification accuracies (%, mean ± std over 20 runs) on the Cambridge and Northwestern datasets.

Method	Accuracy	
	Cambridge	Northwestern
SPD	98.20%±0.58%	47.95%±2.08%
Optical flow histogram(ofh)	91.09%±1.33%	96.75%±0.51%
spd + ofh	**99.31%±0.39%**	**97.23%±0.54%**

On the Cambridge dataset, the SPD feature alone achieves a high accuracy of 98.20%, indicating that spatial information is largely sufficient for this dataset. The OFH feature extracted from 6 keyframes also performs well, reaching 91.09%. By combining SPD and OFH features, the accuracy is further improved to 99.31%, demonstrating a complementary effect between spatial and temporal representations.

In contrast, on the Northwestern dataset, the SPD feature alone performs poorly, achieving only 47.95% accuracy. However, the OFH feature extracted from 16 keyframes significantly improves the performance to 96.75%, highlighting the importance of temporal motion information. The combination of SPD and OFH features further boosts the accuracy to 97.23%, achieving the best overall performance.

#### Error analysis and methodological justification.

**Fig 8:**
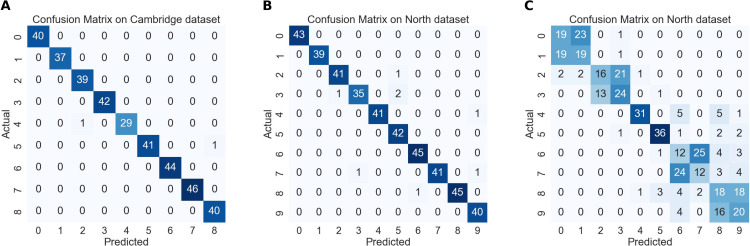
Confusion matrices for analyzing the classification results: (A) Combined Features – Cambridge, (B) Combined Features – Northwest, (C) SPD Features – Northwest.

To better understand the performance differences observed in the ablation experiments, we analyze the confusion matrices shown in Fig 8. Subfigures 8 (A) and (B) present the results of the combined SPD + OFH features on the Cambridge and Northwestern datasets, respectively, while subfigure 8 (C)shows the SPD-only results on the Northwestern dataset.

The combined features achieve strong performance, indicating that integrating spatial and temporal information captures complementary cues. Specifically, SPD features primarily capture spatial relationships, while OFH features encode temporal dynamics, enabling the model to distinguish directional motion patterns. In contrast, the SPD-only confusion matrix reveals systematic misclassification patterns (e.g., “move right” vs. “move left,” “rotate up” vs. “rotate down”), demonstrating that SPD features alone cannot capture temporal ordering due to their covariance-based representation, which ignores temporal information. This explains the poorer performance of SPD alone, particularly on the Northwestern dataset which relies heavily on temporal information.

Overall, this analysis provides insight into why the individual components perform differently and supports the methodological choice of integrating SPD and OFH features, highlighting their complementary roles and validating the design of our feature integration strategy.

Our evaluation on the Cambridge and Northwestern datasets achieved recognition accuracies of 99.31% ± 0.39% and 97.23% ± 0.54%, as detailed in Table 5 (Cambridge) and Table 6 (Northwestern). These results demonstrate that the proposed approach achieves strong and competitive performance compared to existing methods, and notably surpasses previously reported results on the Cambridge dataset. The observed performance gains can be attributed to the effective integration of spatial features modeled on the SPD manifold with complementary temporal motion features. To provide context, we briefly analyze the main ideas behind the compared algorithms and discuss their respective limitations.

**Table 5:** Accuracy comparison with existing methods on the Cambridge dataset.

Cambridge	Method	Accuracy
Wong et al. [47]	Sparse Bayesian Classifier	44%
Niebles et al. [48]	Spatial-Temporal Words	67%
Kim et al. [46]	Tensor Canonical Correlation Analysis	82%
Kim et al. [49]	Canonical Correlation Analysis	82%
Liu et al. [23]	Genetic Programming-Evolved Descriptors	85%
Lui et al. [50]	High Order Singular Value Decomposition	88%
Lui et al. [51]	Tangent Bundle	91%
Wong et al. [52]	Probabilistic Latent Semantic Analysis	91.47%
Sanin et al. [53]	Riemannian Covariance Descriptors	93%
Baraldi et al. [54]	Dense Trajectories with Hand Segmentation	94%
Zhao et al. [55]	Information Theoretic	96.22%
Uke et al. [56]	Hybrid Spatiotemporal-SURF Feature Extraction	97.9%
Tang et al. [12]	Euclidean Space Feature Fusion	98.23% ± 0.84%
Yu et al. [57]	2D CNN with Fractional Optical Flow and Feature Fusion	98.62%
Our method	Hybrid SPD-Euclidean Feature Integration	**99.31% ± 0.39%**

**Table 6:** Accuracy comparison with existing methods on the Northwestern dataset.

Northwestern	Methods	Accuracy
Liu et al. [23]	Genetic Programming-Evolved Descriptors	96.1%
Shen et al. [24]	Motion Divergence Field Descriptor	95.8%
Uke et al. [56]	Hybrid Spatiotemporal-SURF Feature Extraction	96.89%
Tang et al. [12]	Euclidean Feature Fusion	96.89% + 1.08%
Yu et al. [57]	2D CNN with Fractional Optical Flow and Feature Fusion	**97.64%**
Our method	Hybrid SPD-Euclidean Feature Integration	97.23% ± 0.54%

Wong et al. [47] developed a method that constructs a motion gradient orientation image from raw video data, transforming it into a motion feature vector for classification using a sparse Bayesian classifier. Meanwhile, Niebles et al. [48] focused on spatial-temporal word representation derived from detected interest points, creating a sparse representation of video sequences; however, this approach may overlook broader spatial information. Kim et al. [46,49] expanded Canonical Correlation Analysis (CCA) to multiway data arrays for robust spatiotemporal pattern recognition, bypassing the need for explicit motion estimation. Liu et al. [23] utilized Genetic Programming to optimize 3D sequence-processing operators for gesture recognition, where accuracy is contingent upon the quality of the selected operators. Lui et al. [50,51] classified videos using distances on Grassmann manifolds, while Wong et al. [52] enhanced motion recognition through a generative model extending probabilistic latent semantic analysis (pLSA). Sanin et al. [53] utilized covariance descriptors and mapped them to Euclidean space for classification. Baraldi et al. [54] combined dense trajectories with segmentation techniques for feature extraction, while Zhao et al. [55] focused on keyframe selection and local motion feature extraction. Tang et al. [12] fused appearance features from SURF with motion features from SIFT 3D for classification. Shen et al. [24] introduced a method that uses motion divergence fields for hand motion recognition, detecting salient regions with Maximally Stable Extremal Regions and extracting local descriptors to capture motion patterns.

Despite significant advancements in hand gesture recognition, existing methods still exhibit certain limitations that can affect their performance. Many approaches focus primarily on either region-of-interest detection or feature extraction within Euclidean space or Riemannian manifolds, often emphasizing a single type of representation. Although both Euclidean and manifold-based features have demonstrated effectiveness in different contexts, their complementary integration has been relatively less explored in gesture recognition tasks.

In this work, we integrate features derived from both Euclidean space and Riemannian manifolds by leveraging the Log-Euclidean metric to map manifold-based features into Euclidean space. This unified representation enables effective fusion of spatial and temporal information, facilitating improved classification performance while maintaining computational efficiency.

## Conclusion

In this paper, we present an innovative method to enhance hand gesture recognition by integrating spatial features represented on the SPD manifold with temporal features derived from optical flow in Euclidean space. Our method addresses limitations associated with Euclidean space representation, which may inadequately preserve intrinsic geometric relationships and consequently affect classification performance. By leveraging the SPD manifold for spatial feature representation and integrating it seamlessly with optical flow features, we achieved significant advancements in gesture recognition accuracy.

The evaluation of our approach on the Cambridge hand gesture dataset showed outstanding results, achieving an accuracy of **99.31% ± 0.39%**, and achieving performance that is comparable to or exceeds previously reported traditional and neural network–based methods on this dataset. On the Northwestern hand gesture dataset, our approach achieved an accuracy of **97.23% ± 0.54%**, remaining highly competitive with recently reported deep learning–based approaches. These results demonstrate the effectiveness of our framework in capturing both the spatial subtleties and temporal dynamics of hand gestures, while maintaining efficiency and interpretability.

Our method not only preserves the geometric properties of gesture data but also enhances the meaningfulness of feature representations, thereby improving classification performance. These findings highlight the potential of integrating manifold-based spatial features with traditional temporal features for robust hand gesture recognition across diverse applications, including human-computer interaction, virtual reality, and assistive technologies.

Moving forward, further exploration could focus on refining feature fusion techniques, scalability to larger datasets, and adapting our method to real-time applications. By continuing to refine and expand upon these findings, we seek to advance the development of gesture recognition technology and its practical applications.
